# Management of esophageal perforations in infants by endoscopic vacuum therapy: a single center case series

**DOI:** 10.1186/s12876-022-02346-2

**Published:** 2022-06-03

**Authors:** Dominik J. Kaczmarek, Dominik J. Heling, Christian P. Strassburg, David Katzer, Gesche Düker, Joanna Strohm, Andreas Müller, Andreas Heydweiller, Tobias J. Weismüller

**Affiliations:** 1 Department of Internal Medicine I, University Hospital Bonn, Venusberg-Campus 1, 53127 Bonn, Germany; 2 Department for Pediatric Medicine, University of Bonn, Bonn, Germany; 3 Department for General, Visceral, Thoracic and Vascular Surgery, University of Bonn, Bonn, Germany; 4 Department for Neonatology and Pediatric Intensive Care Medicine, University of Bonn, Bonn, Germany; 5 Department for Pediatric Surgery, Sankt Marien-Hospital, Bonn, Germany; 6 Present Address: Department of Internal Medicine - Gastroenterology and Oncology, Vivantes Humboldt Hospital, Berlin, Germany

**Keywords:** Endoluminal vacuum therapy, Insufficiency, Dehiscence, Leakage, Negative pressure therapy (npt), Newborns, Polyurethane foam, Prematurely born infants, Rupture

## Abstract

**Background:**

Endoscopic vacuum therapy (EVT) has become a standard treatment method for esophageal perforations in adults. However, experience with EVT in infants is scarce. In this retrospective case series, we report on four very young infants who were successfully treated with EVT for esophageal perforations of different etiology.

**Methods:**

Four infants were diagnosed with esophageal perforations on day 7, 32, 35 and 159 of life, respectively. The youngest one was prematurely born in the 31^st^ week of pregnancy weighing 980 g only. Three infants had perforations due to foreign body insertion (nasogastric tube or pulling through of percutaneous endoscopic gastrostomy (PEG) tube through the esophagus). One child had an anastomotic dehiscence after Foker’s surgery for atresia. In three children EVT was applied as first-line therapy for perforation, in one child EVT was a rescue therapy due to persisting leakage after surgical closure involving thoracotomy. Depending on the esophageal diameter, either an open-pore drainage film or polyurethane sponge was attached to a single-lumen 8 Fr suction catheter, endoscopically (or fluoroscopically by wire-guidance) placed into the esophagus (intraluminal EVT) and supplied with continuous negative pressure (ranging between 75 and 150 mmHg). The EVT system was exchanged twice per week.

**Results:**

Complete closure of the perforation/leakage could be achieved in all four infants (100%) after 22 days of continuous EVT (median value; range 7–39) and 4.5 EVT exchanges (median value; range 1–12). No serious adverse events occurred.

**Conclusions:**

EVT is an effective and safe addition to our therapeutic armamentarium in the management of esophageal perforations irrespective of its etiology. Here we prove the feasibility of EVT even in very young infants. The use of an extra thin vacuum open-pore drainage film is helpful to cope with the small esophageal diameter. EVT settings and exchange rates similar to those known from adult treatment were used.

## Background

In adults, potentially life-threatening esophageal perforations and leakages can occur spontaneously as Boerhaave’s syndrome, as late manifestation of advanced esophagitis, consequence of trauma or foreign body ingestion or can be frequently encountered as complication of surgical or endoscopic interventions. Esophageal leakages are, however, not only a domain of adult medicine but can be also seen in infants with prematurely born infants affected predominantly [1–8]. While there have been case reports of spontaneous esophageal perforations, they are mainly iatrogenic following procedures like resuscitation, difficult intubation or insertion of a nasogastric feeding tube. Many cases can be managed conservatively, i.e. the perforation will heal spontaneously while infants are kept on broad spectrum anti-infective agents and nothing per os (NPO) [3]. Still, full-thickness perforations of the esophagus are a life-threatening condition and some of the infants will develop mediastinitis and require surgical therapy to close the perforation [6, 7].

Endoscopic vacuum therapy (EVT) has become a standard treatment of perforations or anastomotic dehiscences (insufficiencies), mainly in the upper gastrointestinal (GI) tract or the rectum, since it was first described by Weidenhagen 2003 [8–16]. This method is derived from vacuum-assisted closure (VAC) therapy of external wounds, as introduced by Argenta and Morykwas in 1997 [17]. EVT within the GI tract involves a polyurethane sponge or a special open-pore film, connected to a suction catheter, which is endoscopically placed either into the lumen of the GI tract, thus covering the perforation site (intraluminal EVT), or inserted into the perforation site itself, i.e. outside of the mucosa-lined space (intracavitary EVT) [10, 18]. The principle of EVT is to create a negatively pressurized compartment at the perforation site providing local drainage of fluids and inducing granulation of the infected wound area, eventually resulting in defect closure [16]. The applied negative pressure (i.e. the “vacuum”) causes a collapse of the surrounding tissue thus sealing it and impeding pressure equalization. While EVT has become a widely accepted first-line therapy for esophageal perforations/leakages of different origin in adults, experience on EVT in infants is still scarce. In 2018, a first case of EVT in an 8 year-old infant was reported by Fraga et al. [19]. Also in 2018, Manfredi et al. reported on a series of 17 children with esophageal atresia who were treated with EVT for esophageal perforations [7]. The success rate was 88%. Less promising results have been lately reported by Ritz et al. [20]: In all five infants, EVT alone was not sufficient and required additional treatment (surgery or insertion of a suction catheter).

In this present retrospective case series, we report on four very young infants who were successfully treated with EVT in our endoscopy unit (7, 32, 35 and 159 days old when diagnosed with perforation/leakage). In one of these infants, prematurely born in the 31^st^ week of pregnancy and weighing 980 g only, EVT was begun on day 24 of life. To our best knowledge, this is the youngest patient up to date in whom EVT was successfully performed.

## Methods

### Patient cohort

In this retrospective case series, we report on four infants who were treated with EVT for esophageal leakages in our endoscopy unit between May 2019 and January 2021. Outcome measures were successful closure of the defect, endoscopy-associated complications, mortality and the persistence or recurrence of a leakage despite endoscopic therapy.

### Endoscopic vacuum therapy

**Fig. 1:**
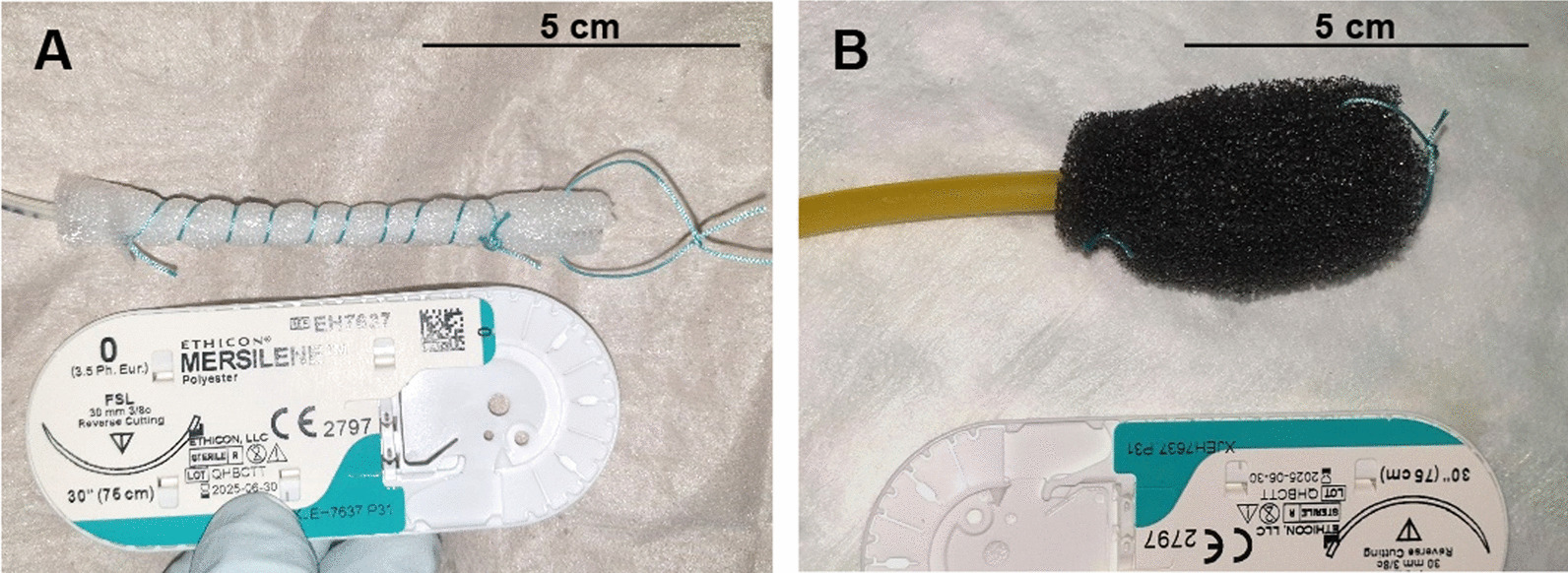
Depending on the diameter of the esophagus, either an open-pore film **A** or an open-pore polyurethane sponge **B** is individually shaped by the endoscopist to cover the perforation site and attached to the tip of an 8 Fr suction catheter by sutures. The film/sponge is then placed into the esophagus. The other end of the catheter is connected to a vacuum pump which applies continuous suction to the perforation site. Suture packages serve as size reference

EVT was performed according to previous reports [10, 18, 21]. Depending on the perforation/leakage size and diameter of the esophagus, either a thin open-pore drainage film (EVT film) (Suprasorb CNP drainage film, Lohmann & Rauscher, Neuwied, Germany) or an open-pore polyurethane sponge (EVT sponge) (V.A.C. Granufoam Dressing Medium, Acelity, Wiesbaden, Germany) was individually shaped by the endoscopist and attached to the tip of a single-lumen 8 Fr suction catheter (EVT catheter) by sutures (Mersilene Polyester, 0/3.5Ph.Eur., Ethicon) as shown in Fig. 1. If necessary, additional suction holes were cut into the catheter to distribute suction to the EVT film/sponge at its full length. Due to the small esophageal diameter in infants, we decided to individually shape the EVT sponge according to our needs rather than deploy a readily manufactured sponge system, as commercially available for EVT. In case of EVT films, there are no commercially available ready-to-use devices, so these must be always shaped individually by the endoscopist. The EVT film/sponge was then perorally guided into the esophagus with a nasal gastroscope (EG-580NW, 5.9 mm, Fujifilm) and carefully positioned under endoscopic view so as to cover the complete perforation site (intraluminal EVT)[10]. The EVT catheter was connected to a vacuum pump (V.A.C. VeraFlo, v.a.c.ulta, Negative Pressure Wound Therapy Unit, Dublin, Ireland) and continuous suction was applied. Suction intensity (negative pressure between 75 and 150 mmHg) was chosen at the endoscopist’s discretion following their experience and pre-existent data from EVT in children [7]. Once firm position of the EVT film/sponge within the esophagus under suction was confirmed, the endoscope was retrieved and the EVT catheter fixed to the infant’s cheek by a tape. EVT sponges can adhere very tightly to the tissue or become non-functional due to blockage caused by mucus. This is why the EVT film/sponge was exchanged twice per week. For EVT exchange, we first confirmed the correct position of the inserted EVT film/sponge endoscopically. Suction was then discontinued and the EVT film/sponge perorally removed. Removal of the EVT film/sponge went easily and required no specific pre-treatment (e.g. flushing of the EVT film/sponge). The perforation site was inspected and assessed for size; if necessary, the wound was cleaned of debris and fibrin and rinsed with NaCl 0.9%. Small aspiration nubs at the perforation site and signs of tissue proliferation, i.e. small granulation tissue nodules, proved the effectiveness of ongoing EVT. Suction intensity was individually adapted (increased) at the endoscopist’s discretion if the tissue response to EVT was considered insufficient. A new EVT film/sponge was then attached to a new suction catheter and reinserted into the esophagus as described above. EVT was discontinued when complete wound closure had been achieved. All endoscopic procedures were performed by a team of experienced interventional gastroenterologists. EVT exchanges were performed under general anesthesia conducted by a team of pediatricians and/or anesthesiologists. For the time of ongoing EVT, i.e. between exchanges, general anesthesia was maintained if primarily needed for other reasons.

### Data presentation

Data are presented as median values with range or, where applicable, as percentage of all patients.

## Results

### Patient characteristics and diagnosis of leakage

Four infants with esophageal leakages of different etiology were presented to our endoscopy unit for endoscopic assessment and therapy. Patient characteristics are given in Table 1.

**Table 1:** Patient characteristics

Infant no.	Sex (m/f)	Birth	Weight at birth (g)	Diagnosis	Age at manifestation of perforation/leakage (days)	Age at initiation of EVT (days)	Weight at initiation of EVT (g)	Mechanism leading to esophageal perforation/leakage
1	m	Prematurely in 31^st^ week of pregnancy (31 + 0)	980	Healthy	7	24	1500	Insertion of feeding tube
2	f	Prematurely in 36^th^ week of pregnancy (36 + 6)	2500	Esophageal atresia type II according to Vogt	159	161	6300	Dehiscence of the esophago-esophageal anastomosis following Foker’s procedure
3	m	Prematurely in 35^th^ week of pregnancy (35 + 5)	2770	Complex syndromic disease with impairment of oral nutrition	32	32	3200	Pulling through of PEG tube (PEG size 15 Fr)
4	f	In 39^th^ week of pregnancy (39 + 5)	3140	Complex syndromic disease with impairment of oral nutrition	35	35	3306	Pulling through of PEG tube (PEG size 9 Fr)

*EVT* endoscopic vacuum therapy, *F* female, *m* male, *PEG* percutaneous endoscopic gastrostomy

**Fig. 2:**
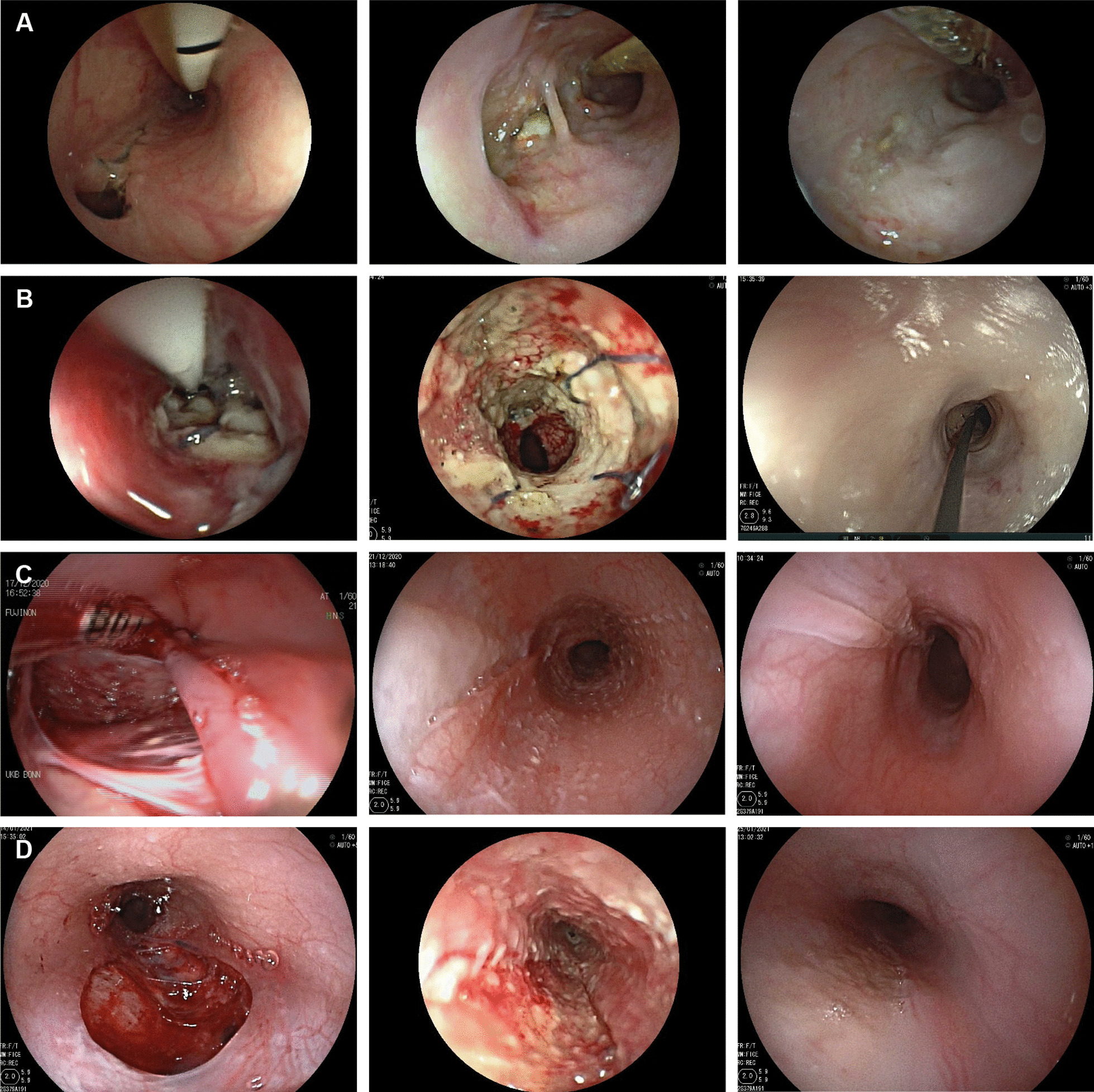
**A** *Infant “1”. Left:* Esophagus on first endoscopic encounter with visible partial suture dehiscence. *Middle:* Day 28 of endoscopic vacuum therapy (EVT). The suture had torn open completely shortly after EVT initiation. *Right:* Day 39 of EVT. Complete defect closure achieved. Scar tissue visible. **B** *Infant “2”. Left:* Esophagus on first endoscopic encounter. Dehiscence of the esophageal anastomosis, involving two thirds of the circumference. *Middle:* Day 20 of EVT. Fibrin and necrotic tissue (grayish) and remainders of surgical sutures (purple) can be seen. Granulation tissue nodules (reddish and sanguineous) are considered as proof of EVT effectiveness. *Right:* 14 days after discharge from EVT. The leakage has completely healed but a stenosis has developed. **C** *Infant “3”. Left:* Perforation site a few hours after the percutaneous endoscopic gastrostomy (PEG) procedure. *Middle:* Day 4 of EVT. EVT has left a regular pattern of aspiration nubs. *Right:* 14 days after discharge from EVT. The defect is completely healed. Scar tissue can be seen. **D** *Infant “4”. Left:* Perforation site a few hours after PEG procedure. *Middle:* Day 4 of EVT with visibility of granulation tissue and aspiration nubs. *Right:* Day 11 of EVT with complete defect closure

Infant “1” (male) was prematurely born in the 31st week of pregnancy weighing 980 g. On day 7 of life the infant was diagnosed with an esophageal perforation, probably caused by insertion of a feeding tube: radiography had shown a strong leakage of contrast agent into the mediastinum. The infant underwent thoracotomy as the perforation would not close spontaneously, and the defect (15 mm in diameter) was closed by sutures. However, the leakage persisted, and the infant was presented to our endoscopy unit 17 days after first manifestation of the perforation, aging 24 days and weighing 1500 g at that time, for further treatment. The defect size at the suture site was 3 mm (Fig. 2A).

Infant “2” (female) was diagnosed with esophageal atresia type II according to Vogt [22] and had to undergo surgery with thoracotomy, gastrostomy and esophageal anastomosis according to Foker’s technique [23]. A dehiscence of the esophageal anastomosis was later assumed and endoscopically confirmed on day 159 of life (weight 6300 g), involving two thirds of the anastomotic circumference (Fig. 2B).

Infant “3” (male) and “4” (female) had complex syndromic diseases comprising an impairment of oral nutrition and had to undergo the percutaneous endoscopic gastrostomy (PEG) procedure at the age of 32 and 35 days (weighing 3200 g and 3306 g, respectively). In both infants, pulling of the PEG tube (sized 15 Fr and 9 Fr, respectively) through the esophagus led to an esophageal perforation of 6 and 4 cm in length, respectively, as seen by the pediatric endoscopist immediately following PEG insertion. Both infants were presented to our endoscopy unit on the same day for further assessment (Fig. 2C and D).

### Endoscopic vacuum therapy

All 4 children received EVT to treat the leakage. EVT data are summarized in Table 2. In infants “2”, “3” and “4”, EVT was chosen as first-line therapy to close the defect while in infant “1” EVT was started as rescue therapy, after surgical treatment had proven unsuccessful. In all four children EVT was started with an EVT film due to the small esophageal diameter, in infants “1” and “2” EVT films were later on exchanged by larger EVT sponges to exert more debriding and granulation forming. In infant “1” the initial EVT film placement had to be done fluoroscopically by wire guidance (Radifocus Guide Wire M Stiff type, Terumo Corporation, Japan) because the post-operative esophagus was too narrow for endoscopic guidance even with a 5 mm nasal endoscope. All other EVT placements in all infants were performed with a nasal gastroscope (diameter 5.9 mm). In infant “2” the anastomotic dehiscence came along with a stenosis. Hence, for EVT initiation and for the first 4 EVT exchanges (out of 7), the anastomosis had to be dilated with biliary dilation balloons (Fusion Titan Biliary Dilation Balloon, Cook Medical, Ireland, increasing sizes from 4 to 10 mm) prior to insertion of the EVT film/sponge. In infants “1” and “2” a negative pressure of 75 mmHg with low intensity was chosen to begin EVT. However, the formation of granulation tissue at this low pressure proved to be insufficient, and therefore negative pressure was stepwise increased to 150 mmHg (high intensity) and 125 mmHg (medium intensity), respectively. Also, in infant “1” EVT exchange sessions were increased from 2 to 3 times per week. In patients “3” and “4” a negative pressure of 100 mmHg with medium intensity was chosen for the entire EVT. Infant “1” was nourished through a percutaneous jejunostomy during ongoing EVT, while all other infants were kept on nil per os (NPO). Infant “2”, however, had a nasogastric feeding tube, used for drug administration only.

**Table 2:** Synopsis of endoscopic vacuum therapy (EVT) data

Infant no.	Initial size of perforation	Begin EVT (days after first diagnosis of esophageal perforation/leakage)	EVT system (film/sponge)	No. of EVT film/sponge exchanges	Duration of EVT (days)	Additional endoscopic therapy / EVT features	Initial EVT pressure (mmHg)	Initial EVT pressure intensity	EVT settings during ongoing EVT	Successful closure at the end of EVT	Adverse events	Outcome
1	3 mm	17	EVT film, later sponge	12	39	First EVT placement was fluoroscopically by wire guidance	−75	Low	−150 mmHg, high intensity	Yes	None	Alive until follow-up, no recurrence of defect
2	2/3 of circumference of anastomosis	2	EVT film, later sponge	7	32	Balloon dilation (increasing from 4 to 10 mm) of anastomosis prior to EVT insertion (on EVT initiation and for first 4 EVT exchanges)	−75	Low	−125 mmHg, medium intensity	Yes	Stenosis of anastomosis	Alive until follow-up, no recurrence of defect
3	6 cm	0	EVT film	1	7	None	−100	Medium	Unchanged	Yes	Gastric ulcer	Alive until follow-up, no recurrence of defect
4	4 cm	0	EVT film	2	11	None	−100	Medium	Unchanged	Yes	None	Alive until follow-up, no recurrence of defect

### Outcome

Successful defect closure was achieved in all four infants after a median EVT duration of 22 days (range 7–39) comprising 4.5 EVT film/sponge exchanges (range 1–12). All infants survived until last follow-up (median 8 months, range 3–21) and there was no recurrence of leakage. Infant “2” developed an anastomotic stenosis after EVT and had to undergo four sessions of balloon dilation on days 14, 21, 28 and 35 after discharge from EVT (Fusion Titan Biliary Dilation Balloon, Cook Medical, Ireland, increasing sizes from 6 to 10 mm). In infant “3”, an ulcer developed in the gastric antrum during ongoing EVT, most probably caused by contact with the PEG tube. No other EVT-associated adverse events were recorded.

## Discussion

Esophageal perforations, irrespective of their etiology, pose a vital threat to patients [2, 3, 6]. There has been much controversy about the optimal therapeutic approach in case of perforation in infants [7, 19]. While many cases can be managed conservatively, i.e. administration of broad-spectrum antibiotics and keeping the infant on NPO, some cases will require high-risk surgical interventions associated with high mortality rates [2, 3, 6]. EVT on the other hand has become a standard procedure for treatment of esophageal perforations and leakages in adults and is a low-risk and easy-to-perform alternative to (thoracic) surgery [24–28]. In the present case series, we confirm the feasibility of EVT in infants. The procedure was adopted from adult medicine and we found that similar EVT settings have to be applied to achieve defect closure, i.e. similar negative pressure values and EVT exchange frequencies. Thus, our data confirm previous reports from Fraga et al. and Manfredi et al. for cases of newborns and very young infants [7, 19]. Lately, Ritz et. al reported on five children with esophageal perforations (median age 3.4 years, the youngest child being 7 months old) [20]. In all five children, EVT helped to significantly reduce local and systemic inflammation and facilitated further treatment, although EVT alone was not sufficient to achieve complete healing of the defect: while 1 child required surgery, the other 4 children healed under subsequent therapy with a suction catheter. One possible explanation could be that Ritz et al. used longer intervals between EVT exchanges and applied lower suction values (100 cmH_2_O being equal to 73.6 mmHg). In our experience (gained from infant “1”), suction values can be stepwise increased if the healing process is lacking and the EVT system can be exchanged more frequently. Also, in the report by Ritz et al. completion of defect closure was achieved by a suction catheter (100 cmH_2_O) in 4 out of 5 children, which might be considered as another form of vacuum therapy. Like in the report by Ritz et al., most of the children in the reports by Fraga et al. and Manfredi et al. were older (median age 24 months, youngest child 3 months) and Manfredi et al. reported on a distinct group of children suffering from esophageal atresia. The novelty of our case series is doubtless the very young age (24, 32, 35 and 161 days of life on initiation of EVT). The youngest infant was prematurely born in the 31st week of pregnancy with a birth weight of 980 g only: on initiation of EVT, the infant was 24 days old weighing 1500 g, the esophagus being too narrow to be passed with a nasal gastroscope. For this reason, initial EVT film placement had to be done fluoroscopically by wire guidance. To our best knowledge this technical approach has not been yet described in EVT.

There are, however, some differences to EVT in adults. Firstly, the EVT catheter can be placed perorally and not transnasally, which in our opinion facilitates EVT placement and EVT exchanges and prevents contact lesions within the nose. In adults, peroral EVT catheter placement will usually not be tolerated by the patient. Secondly, the infant’s esophagus has a much smaller diameter, so EVT placement can be more cumbersome and requires the use of a nasal gastroscope or even fluoroscopic wire guidance (as described in case “1”). In all four cases, we used thin 8 Fr suction catheters and began therapy with EVT films (instead of sponges) because EVT films can be shaped much thinner. Thus, owing to the availability of thin open-pore drainage films, the use of which to our best knowledge has not been yet described in infants, EVT becomes technically feasible even in very young infants with small esophageal diameters. However, from our own and other authors’ experience in adults, EVT sponges adhere more strongly to the tissue and for this reason exert higher debriding properties than EVT films, inducing more granulation and eliminating necroses more efficiently [29]. In older wounds containing a lot of debris (like in cases “1” and “2” described here) it is therefore desirable to switch to EVT sponges if technically possible. In adults, EVT placement and exchanges are performed under conscious sedation, but patients usually stay awake during ongoing EVT. Similarly, EVT in infants does not seem to require general anesthesia on principle, and lowest sedation levels possible should be rather sought. Most infants in our cases (“1”, “3” and “4”) were kept on general anesthesia for the time of ongoing EVT, however, in infants “3” and “4” general anesthesia was maintained because of their complex diseases including chronic respiratory insufficiency. In infant “1” the combination of extreme prematurity, little birth weight, status post thoracic surgery and the imminent dislocation risk of the EVT catheter added up to the decision to maintain general anesthesia. Finally, enteral nutrition is an important issue in ongoing EVT, especially given that EVT can last up to several weeks. While patients must be NPO because oral nutrition would contaminate both the perforation site and the film/sponge, thus making EVT ineffective, adults in some cases can be nourished via a nasoduodenal or nasojejunale tube. However, placing a second line for nutritional purposes will be very difficult in an infantile esophagus due to its narrow size or might interfere with the EVT system. Double-lumen feeding tubes on the other hand could help to shape individual EVT film systems that could allow for simultaneous enteral nutrition in future cases, as described by Loske et al. [30, 31].

Owing to its retrospective nature, this report of four more or less independent cases leaves some questions unanswered and makes it difficult to generalize our promising results. Existing data suggests that most infants recover spontaneously after esophageal perforation [3]. Thus, it can be speculated that some of our cases presented here might have also recovered without EVT. On the other hand, cases “1” and “2” describe post-operative situations where an esophageal leakage remained despite or due to surgical therapy, which is why recovery without further specific therapy seemed very unlikely. In cases “3” and “4” the perforation site was very extensive in size (6 and 4 cm) but owing probably to the rapid intervention immediately after perforation, complete closure could be achieved after 7 and 11 days of continuous EVT. Thus, with availability of EVT as a minor invasive and easy-to-perform procedure with a low-risk profile, a watch-and-wait approach after esophageal perforation might be questioned. Though infant “2” developed an anastomotic stenosis after EVT, it can be argued that the stenosis was a consequence of ischemia and local inflammation at the insufficient anastomosis rather than EVT itself. In infant “3”, an ulcer developed in the gastric antrum during ongoing EVT, most probably caused by contact with the PEG tube. Such contact could have been aggravated due to continuous deflation of the stomach, so a causative association with EVT cannot be excluded. Apart from that, no other EVT-associated adverse events were recorded.

Finally, we did not compare EVT with other endoscopic closure techniques, like clip or stent application or insertion of a simple suction catheter. However, several meta-analyses found that EVT in comparison to the use of self-expandable metal stents (SEMS) for esophageal leaks had higher closure rates, shorter treatment times and lower mortality rates [27, 28]. Manfredi et al. in their study also compared EVT with stent placement in infants for esophageal perforation due to atresia therapy: they found EVT to be more beneficial than stent placement [7].

## Conclusions

Our case series provides a proof of principle by showing that intraluminal EVT can be a safe and very effective minimally invasive therapeutic addendum to the management of esophageal perforations and leakages even in newborns or very young infants. Owing to its non-invasive nature and bearing in mind the existing favorable data on EVT in adults, EVT should be taken into consideration as a first-line alternative to more invasive therapeutic approaches. Further studies might help to gain more experience in treating infants with EVT and to establish guidelines as to which cases will profit best from EVT and justify EVT over a watch-and-wait strategy.

## Data Availability

All data generated or analyzed during this study are included in this published article. The datasets used and analyzed during the current study are also available from the corresponding author on reasonable request (Dominik J. Kaczmarek, email: Dominik.Kaczmarek@ukbonn.de).

## Abbreviations

EUS: Endoscopic ultrasound
EVT: Endoscopic vacuum therapy
GI: Gastrointestinal
NPO: Nil per os
PEG: Percutaneous endoscopic gastrostomy
SEMS: Self-expandable metal stents
VAC: Vacuum-assisted closure
